# Mechanistic Insights into Dihydromyricetin: Redox Modulation and Kinase-Mediated Control of Disease Pathogenesis

**DOI:** 10.3390/ijms27104626

**Published:** 2026-05-21

**Authors:** Oluwatoyin Adenike Fabiyi, Ayorinde Victor Ogundele, Sulyman Olalekan Ibrahim, Hassan Ibrahim, Héctor Hernán Silva

**Affiliations:** 1 Department of Crop Protection, Faculty of Agriculture, University of Ilorin, Ilorin 240003, Nigeria; 2 Departamento de Ciencias Basicas, Facultad de Medicina, Universidad de La Frontera, Temuco 4780000, Chile; hector.silva@ufrontera.cl; 3 Department of Industrial Chemistry, Faculty of Physical Sciences, University of Ilorin, Ilorin 240003, Nigeria; 4 Department of Chemistry, Federal University of Lafia, Lafia 950101, Nigeria

**Keywords:** dihydromyricetin, diseases, flavanol, bioactivity, metabolism

## Abstract

Dihydromyricetin (DHM), a naturally occurring flavanonol predominantly found in medicinal plants like *Ampelopsis grossedentata*, has emerged as a promising source of natural antioxidants with multi-target pharmacological activities relevant to drug discovery. DHM exhibits a strong redox-modulating capacity, effectively attenuating oxidative stress and inflammation central drivers of chronic disease pathogenesis. Beyond direct radical scavenging, DHM regulates multiple redox-sensitive and kinase-mediated signalling pathways, thereby influencing key cellular processes involved in disease initiation and progression. This review synthesizes current evidence on the therapeutic potential of DHM, critically evaluating its mechanistic basis and translational prospects, with emphasis on its dual redox-driven and kinase-mediated modes of action. We detail its roles in metabolic disorders such as diabetes, obesity, and liver diseases, neuroprotection, cardio protection, and cancer prevention, focusing on the modulation of critical networks such as AMPK, PI3K/Akt, MAPK, NF-κB, and Nrf2. The interplay between these pathways underpins DHM’s efficacy across disease models. Furthermore, we highlight structure–activity relationship (SAR) analyses and molecular modelling studies that elucidate how the flavanonol scaffold, hydroxylation pattern, and stereochemistry of DHM govern its biological activities and target engagement. Key pharmacokinetic limitations, advances in extraction techniques, bioavailability challenges, and emerging formulation strategies including advanced delivery systems are discussed to address translational hurdles. Despite compelling preclinical data, the clinical translation of DHM remains constrained by limited human studies and incomplete mechanistic resolution. This review underscores the need for integrated pharmacological studies and innovative delivery approaches to translate the multifaceted promise of DHM into viable clinical interventions.

## 1. Introduction

Chronic diseases such as cardiovascular diseases, diabetes, neurodegenerative diseases, and cancer have become the leading causes of morbidity and mortality worldwide, significantly affecting public health. These conditions are primarily driven by oxidative stress and inflammation, which contribute to the progressive damage of tissues and organs, and disrupt normal cellular functions. Oxidative stress arises when there is an imbalance between the production of reactive oxygen species (ROS) and the body’s antioxidant defence systems [1,2]. These ROS, including free radicals, can damage cellular components such as lipids, proteins, and DNA, leading to cellular dysfunction, apoptosis, and aging. At the same time, inflammation, a natural immune response to injury or infection, can become chronic and pathological in many diseases, exacerbating tissue damage and contributing to disease progression [3].

As the understanding of these underlying mechanisms grows, the need for effective therapeutic agents that can modulate oxidative stress and inflammation has become more urgent [4]. Natural compounds, derived from plants, have emerged as viable alternatives due to their multi-target activity, low toxicity, and broad-spectrum efficacy [5]. Flavonoids, a large class of polyphenolic compounds found in fruits, vegetables, and herbs, have attracted considerable attention for their ability to regulate both oxidative stress and inflammatory pathways [6,7]. One such flavonoid is Dihydromyricetin (DHM), a flavanonol isolated from the traditional Chinese medicinal herb *Ampelopsis grossedentata* [8].

Dihydromyricetin (DHM), a naturally occurring flavonoid, is increasingly recognized for its significant therapeutic potential in combating a wide range of diseases. Primarily sourced from the traditional Chinese medicinal plant *Ampelopsis grossedentata*, DHM has gained attention due to its robust antioxidant, anti-inflammatory, anti-cancer, neuroprotective, and hepatoprotective properties [9,10]. As a member of the dihydroflavonol subclass of flavonoids, DHM stands out due to its impressive bioactivity profile, offering a novel avenue for therapeutic interventions in chronic diseases, particularly those associated with inflammation, metabolic disorders, and oxidative stress [11,12].

While DHM’s pharmacological effects have been extensively studied, the underlying mechanisms remain an area of active exploration. A deeper understanding of the molecular pathways through which DHM exerts its benefits is crucial for harnessing its full potential in clinical applications. Central to its therapeutic effects are the modulation of redox-sensitive signalling pathways and kinase-mediated pathways, which play pivotal roles in regulating cellular homeostasis, inflammation, metabolism, and apoptosis [13]. In particular, DHM’s ability to regulate key pathways such as AMPK, PI3K/Akt, and MAPK provides a promising framework for its application in various disease states. By influencing these molecular networks, DHM is capable of mitigating disease pathogenesis at multiple levels, from cellular stress responses to tissue-specific effects [14,15,16].

Despite the promising pharmacological activity of DHM, its clinical application has been significantly hindered by challenges related to its poor bioavailability and chemical instability [17]. As a flavonoid, DHM faces the typical hurdles of low solubility and poor membrane permeability, which limit its therapeutic efficacy when administered orally [18]. Moreover, DHM’s high sensitivity to environmental factors such as light, temperature, and pH exacerbates its instability, further complicating its practical use in therapeutic formulations [19]. Consequently, substantial efforts have been directed towards improving the bioavailability and stability of DHM through innovative drug delivery systems, chemical modifications, and formulation strategies. These advancements are essential for overcoming the limitations that hinder DHM’s translation from laboratory findings to clinical practice.

This review aims to comprehensively assess the therapeutic potential of DHM, with a particular focus on its redox-driven and kinase-mediated mechanisms of action. We will explore the botanical sources of DHM, its occurrence in various plant species, and the chemical profile that underpins its biological activities. Furthermore, we will examine the structural activity relationships (SAR) of DHM, highlighting how its chemical structure influences its bioactivity, as well as the molecular modelling studies that provide insights into its interaction with target receptors. A critical analysis of DHM’s redox-modulating activity will be presented, from its direct scavenging of reactive oxygen species (ROS) to its regulation of redox signalling pathways that influence cellular function and disease progression. Additionally, the review will delve into the kinase-mediated signalling pathways, focusing on the impact of DHM on AMPK, PI3K/Akt, and MAPK pathways, which are integral to cellular metabolism, survival, and inflammation.

In parallel, the review will explore the anti-inflammatory and immunomodulatory mechanisms of DHM, which are central to its therapeutic effects in conditions such as cardiovascular disease, cancer, and neurodegenerative disorders. These properties make DHM a promising candidate for the management of diseases driven by chronic inflammation and oxidative stress. A comprehensive evaluation of the therapeutic applications of DHM will be provided, encompassing its potential in treating metabolic diseases, neurodegenerative conditions, and inflammatory disorders, among others.

In light of the significant challenges surrounding the stability and bioavailability of DHM, we will also review the latest advancements in drug delivery technologies, including nanocarriers and microemulsions, which show promise in enhancing the solubility and stability of DHM. These advanced delivery systems offer a potential solution to the limitations of DHM and provide new opportunities for its clinical application. Furthermore, the safety and toxicological profiles of DHM will be examined, with a particular focus on its long-term use and any potential adverse effects. Translational considerations, such as regulatory challenges and the need for robust clinical evidence, will be discussed to provide a clear pathway for the eventual commercialization of DHM-based therapeutic products.

Finally, the review will highlight the current limitations in DHM research and clinical application, offering a critical perspective on the gaps in knowledge and the challenges that remain. Future perspectives will be provided, emphasizing the need for further research to fully understand the mechanisms of action of DHM, optimize its pharmacokinetic properties, and explore its potential in combination therapies for enhanced therapeutic efficacy. Through this comprehensive analysis, we aim to underscore the vast potential of DHM as a versatile therapeutic agent and provide a roadmap for future studies that will pave the way for its clinical success.

### Literature Search Strategy and Selection Criteria

A comprehensive literature search was conducted to identify relevant studies on DHM and its pharmacological mechanisms. Electronic databases including PubMed, Scopus, Web of Science, and Google Scholar were systematically searched for articles published up to April 2026. The search strategy employed combinations of keywords such as “dihydromyricetin”, “ampelopsin”, “oxidative stress”, “AMPK”, “PI3K/Akt”, “MAPK”, “NF-κB”, “Nrf2”, “NLRP3 inflammasome”, “inflammation”, “neuroprotection”, “metabolic disorders”, and “drug delivery”.

Studies were selected based on relevance to the mechanistic, pharmacological, and therapeutic aspects of DHM. Both in vitro and in vivo experimental studies, as well as clinical and formulation-related investigations, were included. Articles lacking sufficient experimental detail or not directly related to DHM were excluded.

The collected literature was critically analysed and organized thematically to provide an integrated overview of DHM’s redox-modulating, kinase-mediated, and immunomodulatory mechanisms, as well as its pharmacokinetic and translational challenges.

## 2. Botanical Sources, Occurrence, Chemical Profile, and Extraction Strategies

### 2.1. Botanical Sources and Occurrence

Dihydromyricetin, also known as ampelopsin, is a flavonoid predominantly found in the plant *Ampelopsis grossedentata*, which is native to East Asia, particularly in southern China. This plant is traditionally used in herbal medicine and as a tea to treat various ailments, including fevers, coughs, and hepatitis. *Ampelopsis grossedentata* is considered the richest source of DHM, with the content of this bioactive flavonoid reaching up to 35% in the tender stems and leaves of the plant [8,17].

In addition to *Ampelopsis grossedentata*, DHM has been isolated from other plant species, such as *Hovenia dulcis* (Japanese raisin tree) [20] and *Cedrus deodara* (Himalayan cedar) [21], both of which are also used in traditional medicine. Furthermore, DHM is present in various fruits, berries, and vegetables, contributing to its dietary intake. The versatile occurrence of DHM in plants with medicinal and dietary significance underscores its potential as a widely accessible bioactive compound [22,23].

### 2.2. Chemical Profile

DHM is a flavonoid belonging to the dihydroflavonol subclass, with a molecular structure characterized by hydroxylated aromatic rings and a benzopyranone skeleton. The chemical formula of DHM is C_15_H_12_O_8_, and its molar mass is 320.25 g/mol. Structurally, DHM is a 2,3-dihydroflavonol, with hydroxyl groups at multiple positions on the flavonoid backbone [24]. The compound’s bioactivity is strongly influenced by the positioning of these hydroxyl groups, which contribute to its antioxidant and anti-inflammatory properties [25].

One of the key features of DHM’s chemical profile is its ability to chelate metal ions, such as zinc (Zn^2+^), which plays a role in its antioxidant activity. This ability to interact with metal ions enhances its redox properties, enabling DHM to scavenge free radicals and mitigate oxidative stress [26,27]. The pyrogallol moiety in the B-ring of DHM is especially crucial for its antioxidant activity, allowing it to scavenge various reactive oxygen species (ROS), including hydroxyl radicals (^•^OH), superoxide anions (^•^O_2_^−^), and other radicals [28,29].

### 2.3. Extraction Strategies

The extraction of DHM from plant sources, particularly *Ampelopsis grossedentata*, is essential for both research purposes and practical applications in nutraceuticals and pharmaceuticals [24]. Traditional extraction methods, such as Soxhlet extraction, are commonly used but suffer from drawbacks, including long extraction times and low yields. These methods are also not well-suited for large-scale industrial applications due to their inefficiency and high solvent consumption [16].

To improve extraction efficiency, alternative methods have been developed. One such method involves the use of a chelating extraction process, where DHM is chelated with Zn^2+^ to prevent oxidation during extraction. This method not only enhances the stability of DHM during extraction but also increases the yield and purity compared to traditional batch extraction. The chelation of DHM with Zn^2+^ reduces the free radical delocalization, preserving the compound’s antioxidant properties. After the chelation process, DHM can be purified by replacing the zinc ion with EDTA (ethylene diamine tetraacetic acid) to obtain a purified form of DHM [30,31].

Additionally, modern techniques such as microwave-assisted extraction (MAE) and high-speed counter current chromatography (HSCCC) have been explored to enhance the efficiency and purity of DHM extraction. These methods allow for faster extraction, with MAE utilizing microwave energy to heat the solvent, thus accelerating the process. HSCCC is particularly effective for the preparative isolation of DHM, offering high purity yields in a shorter time compared to traditional methods [32]. Furthermore, the development of enzymatic processes, such as enzymatic hydrolysis or acylation, has been explored to modify the solubility and bioavailability of DHM, making it more suitable for use in drug formulations [33].

The botanical sources and occurrence of DHM highlight its widespread presence in plants with medicinal and nutritional value, particularly *Ampelopsis grossedentata*. The chemical profile of DHM, characterized by its hydroxylated flavonoid structure and redox-active moieties, underpins its diverse biological activities, including antioxidant, anti-inflammatory, and potential anti-cancer effects. However, challenges related to its extraction and poor bioavailability necessitate the development of improved extraction strategies and formulation approaches to maximize its therapeutic potential. As research progresses, the ongoing refinement of extraction techniques and the exploration of innovative delivery systems will likely pave the way for DHM’s broader clinical applications.

## 3. Structure–Activity Relationships and Molecular Modelling

The structure–activity relationship (SAR) of DHM holds the key to unlocking its full therapeutic potential. As a flavonoid, DHM exhibits remarkable bioactivity, and its structural features are central to the compound’s efficacy. The hydroxylation pattern plays a crucial role, particularly at the 3′, 4′, and 5′ positions on the B-ring, which have been shown to significantly contribute to DHM’s antioxidant properties [34]. These hydroxyl groups enable DHM to interact with metal ions like zinc (Zn^2+^), enhancing its redox properties and enabling it to scavenge harmful free radicals, such as superoxide anions and hydroxyl radicals. However, this very modification also makes DHM more polar, which may limit its lipophilicity and membrane permeability. Thus, researchers have suggested that methylation or acetylation at these positions could potentially balance the antioxidant effects while improving the compound’s ability to cross biological membranes [24,25].

Moving beyond the B-ring, the A-ring of DHM is another critical site that affects its biological activity. The hydroxyl group at the 7-position of the A-ring has been associated with kinase modulation, particularly in AMPK and PI3K/Akt pathways, which are key in regulating metabolism and inflammation. Structural modifications to this position, such as methylation or esterification, could increase DHM’s lipophilicity, making it easier to cross the blood–brain barrier or penetrate other barriers that are crucial in the treatment of diseases like neurodegeneration and cancer [35,36].

To further refine the SAR analysis, molecular docking studies have emerged as an invaluable tool. These computational approaches offer a deeper understanding of how DHM’s structural components interact with biological targets. Recent studies have shown that specific hydroxylated derivatives of DHM exhibit enhanced binding affinity to key receptors like NF-κB and PPARγ, which are involved in inflammation and metabolism. These insights into molecular interactions offer promising avenues for optimizing DHM’s therapeutic effects, helping researchers design derivatives that are more potent, selective, and bioavailable [25,35,37].

### 3.1. Key Structural Features and Their Impact on Bioactivity

The key to DHM’s bioactivity lies in its hydroxylation pattern, which influences its ability to interact with various biological targets. The hydroxyl group at the 3-position of the A-ring plays a crucial role in the compound’s antioxidant activity, enabling it to scavenge reactive oxygen species (ROS) [38]. The pyrogallol moiety present in the B-ring is particularly important for its antioxidant capacity, as it facilitates the chelation of metal ions and the direct scavenging of free radicals [39]. This structural feature is thought to be responsible for DHM’s ability to mitigate oxidative stress, a major contributor to the pathogenesis of various chronic diseases.

In addition to its antioxidant properties, the structure of DHM also influences its anti-inflammatory and kinase-mediated effects. The flavonoid’s ability to modulate signalling pathways such as AMPK, PI3K/Akt, and MAPK is crucial for its therapeutic potential in metabolic and inflammatory diseases. The hydroxyl groups on the A-ring and B-ring are involved in the interaction with these pathways, modulating key proteins involved in inflammation, apoptosis, and cellular metabolism [40,41].

### 3.2. Molecular Modelling and Docking Studies

Molecular modelling and docking studies are essential tools in understanding how DHM interacts with its molecular targets. Recent computational studies have provided valuable insights into the binding modes and mechanisms of DHM in relation to key biological targets, such as kinases and transcription factors [42]. For example, DHM has been shown to interact with the catalytic domains of peroxisome proliferator-activated receptor γ (PPARγ), a nuclear receptor involved in the regulation of glucose and lipid metabolism. Molecular docking studies suggest that DHM binds to the PPARγ receptor, potentially acting as an agonist and modulating its activity to regulate metabolic processes [14,43,44].

Further docking studies have shown that DHM also interacts with various components of the NF-κB signalling pathway, which is crucial for mediating inflammation and immune responses. Inhibition of the IKK complex, a key regulator of NF-κB activation, has been observed upon DHM binding [45,46]. This interaction prevents the phosphorylation and degradation of IκBα, thereby suppressing the nuclear translocation of p65, a critical step in the activation of NF-κB target genes involved in inflammation [47]. These findings suggest that DHM’s anti-inflammatory effects are mediated, at least in part, through the modulation of NF-κB signalling, and molecular modelling has provided insights into the precise binding interactions responsible for this effect.

### 3.3. Stereoisomerism and Its Role in Bioactivity

**Figure 1:**
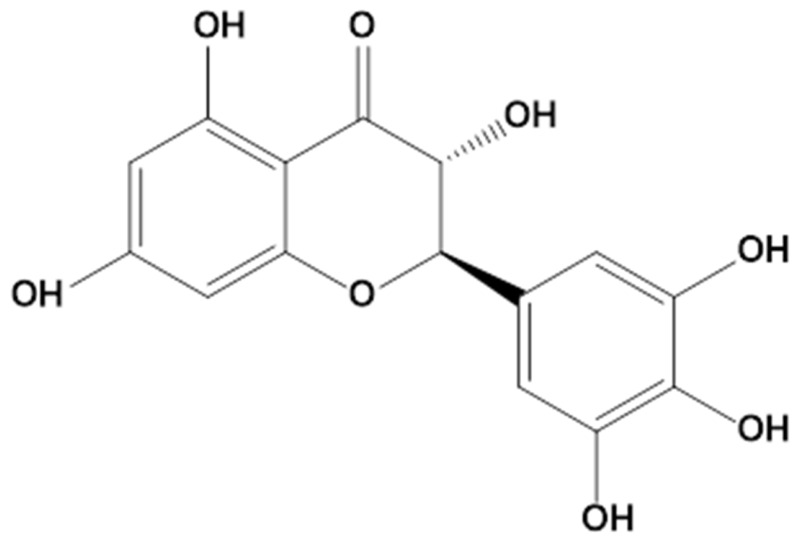
Chemical structure of Dihydromyricetin.

The stereoisomerism of DHM further complicates the structure–activity relationship, as different stereoisomers may exhibit distinct bioactivities; structure of DHM is shown in Figure 1. DHM exists in four theoretical stereoisomers due to the presence of two chiral centres at positions 2 and 3 of the flavonoid backbone [48,49]. Among these, the homochiral (+)-DHM (2R,3R configuration) has been shown to possess superior bioactivity compared to the racemic mixture (±)-DHM [44]. In particular, the (+)-DHM isomer exhibits stronger antioxidant and anti-inflammatory effects, suggesting that the stereochemistry of DHM plays a critical role in determining its pharmacological properties [16,33].

Molecular modelling studies have further elucidated how the stereoisomers of DHM interact with biological targets. The distinct configuration of the hydroxyl groups in the (+)-DHM isomer likely enhances its binding affinity to receptors and enzymes involved in redox regulation and inflammation. This stereochemical advantage may explain the superior therapeutic effects observed with (+)-DHM in certain biological assays [16,50].

### 3.4. Optimizing DHM for Therapeutic Applications

The structural features and stereochemistry of DHM provide a basis for optimizing its therapeutic applications. Understanding the SAR of DHM allows researchers to identify key structural modifications that could enhance its potency, bioavailability, and selectivity for specific molecular targets [24,51]. For example, enhancing the solubility and stability of DHM through chemical modifications could increase its bioavailability and therapeutic efficacy [52]. Moreover, targeting specific kinases or transcription factors through structure-based drug design could lead to the development of more potent and selective DHM derivatives for the treatment of diseases such as cancer, diabetes, and cardiovascular disease [34].

**Figure 2:**
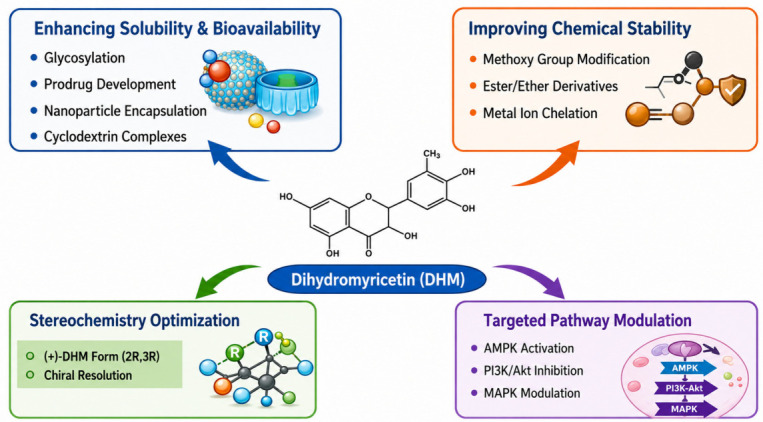
Optimization Strategies for Enhancing the Therapeutic Potential of DHM.

The structure–activity relationship of dihydromyricetin reveals the critical role of its hydroxylation pattern and stereochemistry in determining its bioactivity [24]. Molecular modelling and docking studies have provided valuable insights into how DHM interacts with key biological targets, such as kinases, transcription factors, and receptors, to exert its therapeutic effects [53]. The ability of DHM to modulate redox pathways and kinase signalling networks is central to its anti-inflammatory, anti-cancer, and metabolic regulatory properties [54]. Understanding these interactions through computational studies is essential for optimizing DHM as a therapeutic agent and for guiding the development of new derivatives with enhanced efficacy and selectivity. As research continues to unveil the molecular mechanisms underlying DHM’s effects, it holds great promise as a versatile therapeutic agent for a wide range of diseases (Figure 2).

This infographic outlines key structural optimization strategies for DHM, focusing on enhancing solubility, stability, and bioavailability, optimizing stereochemistry for target specificity, and modulating critical signalling pathways to improve its therapeutic efficacy. These strategies are essential for overcoming the current challenges limiting DHM’s clinical application in treating chronic diseases.

### 3.5. Proposed Derivatization Strategies

**Figure 3:**
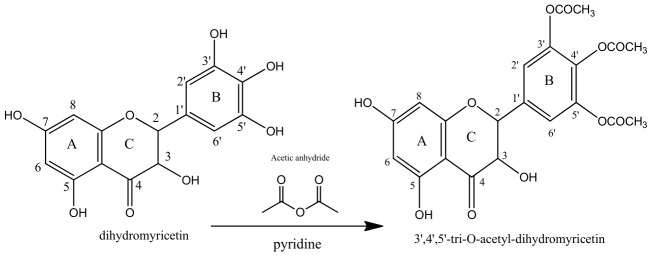
Acetylation Pattern Modifications of Dihydromyricetin.

Several derivatization strategies can be applied to DHM to improve its bioavailability and metabolic stability. These modifications could potentially increase solubility, enhance lipophilicity, and improve interaction with specific biological targets. Below are potential modifications based on SAR analysis:

The 3′, 4′, and 5′ positions on the B-ring of DHM are critical for antioxidant activity but increase polarity, limiting membrane permeability. To address this, acetylation at these positions creates a lipophilic pro-drug (Figure 3). This modification masks the polar hydroxyls to enhance oral bioavailability and cellular uptake; once inside the cell, intracellular esterase’s remove the acetyl groups to restore the compound’s potent antioxidant activity [55].

The figure illustrates potential modifications to the 3′, 4′, and 5′ hydroxyl groups on the B-ring of DHM. Methylation or acetylation at these positions could improve lipophilicity and enhance bioavailability without compromising antioxidant activity, thus aiding in its cellular uptake and oral bioavailability.

#### 3.5.1. Glycosylation

**Figure 4:**
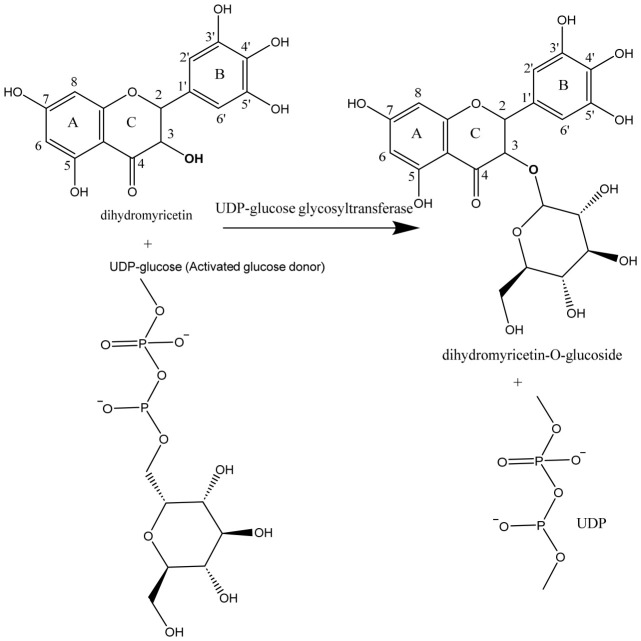
Glycosylation Strategy for Enhanced Bioavailability.

A glycosylation strategy could be employed by attaching sugar moieties to DHM (Figure 4). This would increase water solubility and could potentially improve intestinal absorption. Additionally, glycosylation could make DHM more bioavailable by facilitating its transport across the gut epithelium via active transport systems [56].

The figure depicts a glycosylation modification of DHM by attaching sugar moieties to increase water solubility and improve intestinal absorption. This modification could enhance the compound’s bioavailability by facilitating its active transport across the gut epithelium.

#### 3.5.2. Methylation of the Hydroxyl Groups

**Figure 5:**
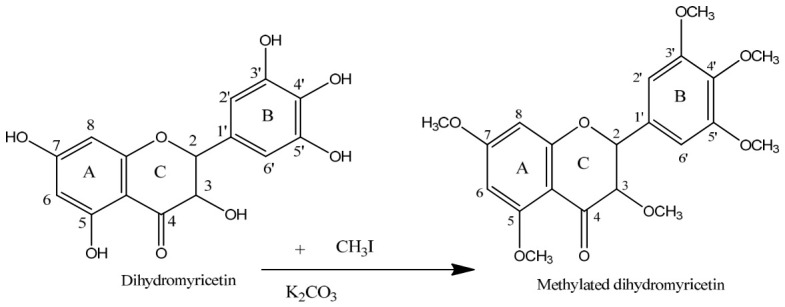
Methylation of Dihydromyricetin.

To enhance lipophilicity and membrane permeability, O-methylation of phenolic hydroxyl groups, including those on the A-ring, B-ring, and C-ring can be explored (Figure 5). Such modifications may improve receptor interactions, metabolic stability, and blood–brain barrier penetration [57].

This figure shows the O-methylation of phenolic hydroxyl groups in DHM. These modifications may increase lipophilicity, improve blood–brain barrier penetration, and enhance receptor affinity, potentially providing therapeutic advantages in conditions requiring enhanced membrane permeability.

#### 3.5.3. Nanoparticle or Liposomal Encapsulation

To overcome the challenges of poor bioavailability, the encapsulation of DHM in liposomes or polymeric nanoparticles could be explored. These delivery systems have been shown to enhance drug solubility, improve stability, and facilitate targeted drug delivery, especially to tissues such as the brain (for neurodegeneration) or tumours (for cancer therapy). Encapsulation can also protect DHM from enzymatic degradation in the gastrointestinal tract, allowing for better systemic distribution [58,59].

#### 3.5.4. Molecular Modelling: Predictions for Modified Derivatives

Once these structural modifications are made to DHM, molecular docking and MMGBSA (Molecular Mechanics Generalized Born Surface Area) calculations should be employed to evaluate the binding affinity of the derivatives to key biological targets, such as kinases (AMPK, PI3K/Akt, MAPK) and oxidative stress-related enzymes (e.g., NADPH oxidase, superoxide dismutase (SOD)). Through these methods, we can predict how these modifications enhance DHM’s therapeutic potential and ensure that the derivatives maintain or improve their efficacy in the treatment of diseases like cancer, diabetes, and neurodegeneration [60].

The MMGBSA (Molecular Mechanics Generalized Born Surface Area) method can be employed to assess the binding energy of DHM derivatives to target proteins. MMGBSA combines molecular mechanics and solvation energy calculations to predict the strength of interactions between DHM derivatives and their targets. The results of these calculations will guide the selection of promising candidates for further in vitro and in vivo testing. The modifications that improve binding affinity, stability, and pharmacokinetic properties can then be prioritized for preclinical evaluation, while drug delivery systems like nanoparticles, liposomes, or polymeric micelles can be used to enhance targeted drug delivery.

## 4. Redox-Modulating Activity: From Direct Scavenging to Pathway Regulation

Maintenance of intracellular redox homeostasis is fundamental for cellular integrity, and disruption of this balance contributes to the development of numerous pathological conditions including cardiovascular disorders, cancer, neurodegeneration, and metabolic diseases [61]. Dihydromyricetin has emerged as a potent modulator of oxidative stress, exerting protective effects through both direct antioxidant activity and regulation of redox-sensitive signalling networks. Rather than functioning solely as a classical radical scavenger, DHM orchestrates multiple molecular processes that collectively restore oxidative balance and prevent oxidative damage.

One of the primary antioxidant mechanisms of DHM involves strengthening endogenous defence systems. Experimental studies have shown that DHM markedly enhances the activity of key antioxidant enzymes such as superoxide dismutase (SOD), catalase, and glutathione-related enzymes, thereby promoting the conversion of superoxide radicals into less reactive species [62]. In models of angiotensin II-stimulated cardiac fibroblasts, DHM treatment significantly decreased intracellular reactive oxygen species (ROS) accumulation and reduced malondialdehyde (MDA), a biomarker of lipid peroxidation [63,64]. These changes were accompanied by a notable increase in total antioxidant capacity and SOD activity, demonstrating the compound’s ability to reinforce cellular antioxidant defences.

In addition to enhancing antioxidant enzyme systems, DHM also suppresses excessive ROS production by modulating oxidative enzyme sources. NADPH oxidases represent a major cellular source of ROS, and evidence indicates that DHM downregulates key oxidase isoforms, particularly NOX2 and NOX4 [65]. In neuronal injury models involving oxygen-glucose deprivation followed by reoxygenation, DHM treatment significantly increased SOD activity while lowering MDA accumulation and reducing the expression of NOX2 and NOX4 [66]. These effects were associated with activation of protective signalling cascades such as the Wnt/β-catenin pathway, suggesting that DHM simultaneously enhances antioxidant defences and limits ROS generation.

Similar regulatory effects have been observed in neuronal systems exposed to ischemic stress. In hippocampal neurons subjected to oxygen-glucose deprivation/reoxygenation injury, DHM suppressed NOX-derived oxidative stress through activation of the Nrf2/HO-1 pathway. This mechanism was accompanied by increased antioxidant enzyme activity and improved cellular redox status, highlighting the central role of Nrf2-mediated transcriptional responses in DHM-induced cytoprotection [66]. Animal studies further confirm the capacity of DHM to mitigate oxidative stress under pathological conditions. In hyperlipidaemic models such as low-density lipoprotein receptor-deficient mice fed a high-fat diet, DHM administration improved systemic oxidative balance by reducing NOX2 expression while restoring the activity of antioxidant enzymes including glutathione, catalase, and SOD [67]. These effects collectively attenuated lipid peroxidation and oxidative damage associated with atherosclerotic progression.

At the molecular level, DHM activates several redox-responsive signalling pathways that coordinate cellular antioxidant responses as presented in Table 1. A key regulatory mechanism involves stimulation of the nuclear factor erythroid-2-related factor 2 (Nrf2) pathway, a master regulator of antioxidant gene expression. DHM promotes Nrf2 activation through multiple processes, including induction of the autophagy adaptor protein p62, which facilitates the degradation of Keap1, the inhibitory partner of Nrf2 [68]. The subsequent release and nuclear translocation of Nrf2 enhance the transcription of downstream antioxidant genes such as heme oxygenase-1 (HO-1). Furthermore, kinase signalling pathways including Akt and ERK contribute to Nrf2 activation by promoting its nuclear localization and transcriptional activity. Beyond Nrf2-dependent regulation, DHM also influences metabolic signalling networks that intersect with oxidative stress control. For instance, modulation of the AMP-activated protein kinase (AMPK) pathway has been implicated in the antioxidant actions of DHM. Through regulation of AMPK activity and downstream targets such as glucose transporter 4 (GLUT4), DHM improves cellular energy balance while simultaneously reducing oxidative stress [69]. This coordinated regulation highlights the close interplay between metabolic homeostasis and redox signalling.

DHM also exhibits notable neuroprotective properties under conditions of hypoxic or oxidative stress. In neuronal models exposed to low-pressure hypoxia, DHM attenuates oxidative injury through activation of the Sirt3–FOXO3a signalling axis [97]. This pathway enhances mitochondrial antioxidant capacity and promotes the expression of genes involved in oxidative defence, thereby preserving neuronal viability and function. In vascular systems, DHM contributes to endothelial protection by regulating nitric oxide (NO) bioavailability. The dimethylarginine dimethylaminohydrolase-1 (DDAH1)/asymmetric dimethylarginine (ADMA)/endothelial nitric oxide synthase (eNOS) pathway plays a critical role in vascular redox balance and endothelial function [98]. DHM has been shown to suppress the expression of microRNA-21, which normally inhibits DDAH1. By relieving this inhibition, DHM increases DDAH1 activity, enhances nitric oxide production, and improves endothelial function [71]. These effects reduce vascular inflammation and lipid metabolic disturbances, thereby lowering the risk of atherosclerotic development.

Protective effects of DHM have also been demonstrated in endothelial cells exposed to oxidative stimuli such as oxidized low-density lipoprotein (ox-LDL). In human umbilical vein endothelial cells (HUVECs), DHM attenuates ox-LDL-induced oxidative injury by reducing ROS accumulation, preventing mitochondrial depolarization, and inhibiting caspase-3 activation [72]. These protective effects are associated with activation of Akt and ERK1/2 signalling, which subsequently stimulate the Nrf2/HO-1 antioxidant pathway. As a result, antioxidant enzymes and anti-apoptotic proteins are upregulated, providing effective cellular defence against oxidative damage. Additional evidence from endothelial oxidative stress models further supports the antioxidant capacity of DHM. In sodium nitroprusside-induced oxidative injury models, DHM pre-treatment markedly reduced intracellular ROS levels and lipid peroxidation while inhibiting apoptosis. The protective effect was mediated through activation of the PI3K/Akt/FoxO3a signalling pathway, which enhances the transcription of antioxidant genes and promotes cellular survival under oxidative conditions [73].

The intrinsic antioxidant capacity of DHM is closely linked to its chemical structure. Structure–activity analyses indicate that the presence of multiple hydroxyl groups within the flavanonol scaffold contributes significantly to its radical scavenging efficiency. In particular, the synergistic interaction between the 3-hydroxyl group on the C-ring and the dihydroxyl configuration on the B-ring provides an effective system for electron donation and radical stabilization. These structural features underpin the strong free radical–neutralizing ability of DHM and contribute to its broad biological activities. Current evidence demonstrates that DHM functions as a multifaceted regulator of oxidative stress. By integrating direct antioxidant activity with modulation of redox-sensitive signalling pathways and enzymatic systems, DHM effectively restores cellular redox balance and protects against oxidative damage associated with diverse pathological conditions.

## 5. Kinase-Mediated Signalling: AMPK, PI3K/Akt, and MAPK Pathways

Protein kinases play pivotal roles in coordinating cellular responses to metabolic stress, inflammation, and environmental stimuli. Increasing evidence indicates that the pharmacological actions of DHM are closely linked to its ability to regulate key kinase-driven signalling networks, particularly those involving AMP-activated protein kinase (AMPK), phosphatidylinositol-3-kinase/protein kinase B (PI3K/Akt), and mitogen-activated protein kinases (MAPKs). Through modulation of these pathways, DHM influences cellular metabolism, survival, autophagy, inflammation, and apoptosis, thereby contributing to its therapeutic potential across diverse pathological conditions.

AMPK functions as a central energy sensor that maintains metabolic homeostasis under conditions of cellular stress [99]. Numerous studies have demonstrated that DHM activates AMPK signalling, triggering downstream events that improve metabolic regulation and cytoprotection. For instance, in models of doxorubicin-induced cardiotoxicity, DHM administration significantly attenuated cardiac injury by activating the AMPK/mTOR signalling axis. This activation suppressed oxidative stress and apoptosis while promoting protective autophagy, thereby preserving myocardial integrity [70]. Similar AMPK-dependent protective mechanisms have been reported in metabolic disorders, where DHM improves glucose homeostasis and enhances insulin sensitivity [80]. The AMPK pathway also contributes to the hepatoprotective actions of DHM in metabolic liver diseases such as non-alcoholic fatty liver disease (NAFLD). NAFLD is increasingly recognized as one of the most prevalent chronic liver disorders worldwide, yet effective pharmacological therapies remain limited. Experimental studies indicate that DHM mitigates hepatic steatosis, inflammation, and oxidative injury through coordinated modulation of AMPK-related signalling pathways, including interactions with NF-κB, MAPK, and sirtuin-dependent mechanisms [84]. These molecular events collectively reduce lipid accumulation and inflammatory responses in hepatocytes.

Beyond hepatic metabolism, AMPK signalling is also implicated in the neuroprotective properties of DHM. In neuronal systems, activation of the AMPK/SIRT1 pathway has been shown to suppress inflammatory signalling and inhibit apoptosis. This pathway regulates transcription factors such as NF-κB and AP-1 while influencing histone acetylation status, thereby contributing to improved neuronal survival and cognitive performance in experimental models of neurodegeneration [74]. In Alzheimer’s disease models induced by amyloid-β peptides, DHM enhances learning and memory while reducing neuronal apoptosis through modulation of the AMPK/SIRT1 signalling cascade and restoration of the balance between pro-apoptotic and anti-apoptotic proteins such as Bax and Bcl-2 [75]. Autophagy is another cellular process strongly influenced by AMPK activation. DHM has been shown to promote autophagic flux in skeletal muscle and neuronal tissues, an effect that contributes to improved metabolic and neurological outcomes. In skeletal muscle cells, DHM enhances insulin sensitivity by activating the AMPK–PGC-1α–Sirt3 signalling axis, which stimulates mitochondrial biogenesis and promotes energy metabolism [80]. Similarly, in neurodegenerative conditions such as Parkinson’s disease, DHM activates AMPK and its downstream target ULK1, thereby promoting autophagy and facilitating the clearance of aggregated α-synuclein proteins [76]. These actions help preserve neuronal viability and reduce disease-associated pathology.

Regulation of glucose metabolism represents another critical outcome of AMPK activation by DHM. Evidence suggests that DHM stimulates glucose uptake through the translocation of glucose transporters such as GLUT1 and GLUT4 to the plasma membrane. This process improves cellular glucose utilization and contributes to overall glycaemic control. In models of insulin resistance, DHM enhances glucose uptake and glycogen synthesis while suppressing lipid accumulation [81]. Mechanistically, these effects involve coordinated activation of AMPK and phosphorylation of Akt, which together regulate key metabolic enzymes and transport systems. In addition, DHM inhibits glycogen synthase kinase-3β (GSK-3β), a kinase implicated in impaired insulin signalling, thereby further contributing to improved metabolic function [82]. In obesity-related insulin resistance models, DHM has also been reported to increase insulin sensitivity by interfering with ERK-mediated phosphorylation of the nuclear receptor PPARγ at serine-273 [81]. This modification promotes adipocyte metabolic reprogramming, enhances glucose uptake, and reduces lipid accumulation. Additional mechanistic studies have identified the phospholipase C (PLC)–CaMKK–AMPK pathway as another important regulatory route through which DHM alleviates inflammation-induced insulin resistance [96]. These findings highlight PLC as a potential molecular target of DHM in metabolic disease management. Despite consistent evidence for AMPK activation, most studies rely on acute in vitro or rodent models, limiting conclusions regarding long-term metabolic regulation and clinical relevance.

The PI3K/Akt pathway represents another major signalling network influenced by DHM. Activation of this pathway plays a crucial role in regulating cell survival, metabolism, and autophagy. In diabetic nephropathy models, DHM modulates the miR-155-5p/PTEN regulatory axis, leading to activation of the PI3K/Akt/mTOR pathway [47]. This signalling cascade promotes autophagy and attenuates renal interstitial fibrosis, thereby protecting kidney function. Similarly, in liver fibrosis models induced by thioacetamide, DHM suppresses inflammation and apoptosis through inhibition of the PI3K/Akt/NF-κB pathway and modulation of transforming growth factor-β1 (TGF-β1) signalling [85]. Activation of PI3K/Akt signalling also contributes to the anti-diabetic effects of DHM. Experimental studies demonstrate that DHM improves insulin resistance by enhancing glucose uptake and promoting intracellular glycogen synthesis under hyperglycaemic conditions. These effects are accompanied by improved glucose consumption and metabolic efficiency [83]. In animal models of diabetes, DHM treatment lowers blood glucose levels and alleviates diabetes-related neuropathic damage through AMPK- and Akt-dependent mechanisms [100]. Clinical observations further suggest that DHM supplementation can improve glycaemic control, lipid metabolism, and renal function in diabetic individuals, supporting its translational potential in metabolic disease management [101]. However, the dual role of PI3K/Akt signalling in both cell survival and pathological proliferation introduces complexity, and context-dependent effects of DHM remain insufficiently resolved.

In addition to AMPK and PI3K/Akt pathways, DHM also modulates mitogen-activated protein kinase (MAPK) signalling, which governs cellular responses to stress and inflammation. The MAPK family includes several kinases, such as ERK, JNK, and p38, that regulate cell proliferation, differentiation, and apoptosis. DHM has been shown to influence MAPK activity in various pathological models. For instance, in renal injury induced by cisplatin, DHM alleviates oxidative stress, inflammation, apoptosis, and ferroptosis through coordinated regulation of Nrf2/HO-1, MAPK, and NF-κB signalling pathways, thereby conferring significant nephroprotective effects [86]. The anti-inflammatory properties of DHM are also partly mediated through modulation of MAPK-dependent signalling networks. In viral infection models, DHM suppresses the production of pro-inflammatory mediators by regulating the TLR4/MyD88/MAPK/NF-κB signalling cascade [46,87]. This mechanism not only attenuates inflammatory responses but also inhibits virus-induced pyroptosis and viral replication, illustrating the broader immunomodulatory potential of the compound.

MAPK signalling additionally contributes to the anticancer activities of DHM. In colon cancer cells, DHM induces apoptosis in a dose- and time-dependent manner through activation of endoplasmic reticulum stress and subsequent stimulation of AMPK and JNK/p38 MAPK pathways [94]. These signalling events trigger the AMPK/MAPK/XAF1 apoptotic cascade, ultimately leading to programmed cell death. Moreover, DHM has been shown to inhibit the invasion and metastatic potential of osteosarcoma cells by disrupting the TNF-α/p38 MAPK/MMP-2 signalling pathway, thereby limiting tumour progression and metastasis [95]. Notably, reported effects on MAPK pathways vary across models and stress conditions, suggesting that DHM may exert context-specific modulation rather than uniform pathway inhibition.

These findings demonstrate that DHM exerts extensive regulatory effects on kinase-mediated signalling pathways. By coordinating the activity of AMPK, PI3K/Akt, and MAPK networks, DHM influences a wide range of cellular processes including metabolism, autophagy, inflammation, apoptosis, and cell survival. This integrated kinase-targeting capacity provides a mechanistic basis for the diverse therapeutic activities of DHM and underscores its promise as a multifunctional natural compound for the management of metabolic, neurodegenerative, inflammatory, and proliferative diseases.

## 6. Anti-Inflammatory and Immunomodulatory Mechanisms

Inflammation is a fundamental biological response to infection, tissue injury, and metabolic imbalance; however, persistent or dysregulated inflammatory signalling drives the progression of numerous chronic diseases [102]. DHM exerts potent anti-inflammatory and immunomodulatory effects through coordinated regulation of innate immune receptors, transcription factors, inflammasomes, and immune cell dynamics.

A central mechanism underlying these effects involves suppression of Toll-like receptor-mediated signalling, particularly the TLR4/MyD88 axis. DHM disrupts the interaction between TLR4 and MyD88 and may additionally interfere with receptor activation through binding to the co-receptor MD2 [90,103]. This inhibition prevents phosphorylation of IκBα and subsequent activation of NF-κB, thereby attenuating transcription of pro-inflammatory genes. As a result, the production of key inflammatory mediators, including TNF-α, IL-6, and IL-1β, is markedly reduced.

Consistent with this, NF-κB signalling represents a major downstream target of DHM. The compound inhibits phosphorylation and nuclear translocation of the p65 subunit, potentially through interaction with critical residues such as Cys46, thereby suppressing expression of inflammatory cytokines and enzymes including inducible nitric oxide synthase (iNOS) and cyclooxygenase-2 (COX-2) [77]. This leads to reduced release of nitric oxide and prostaglandin E2, reinforcing its anti-inflammatory activity [71]. While inhibition of NF-κB is consistently reported, many studies infer pathway suppression from downstream cytokine changes without direct mechanistic validation. DHM also modulates inflammasome activation, particularly the NLRP3 complex, which governs the maturation of IL-1β and IL-18. By inhibiting upstream signalling pathways such as TLR4/Akt/HIF-1α and activating SIRT1-dependent mechanisms, DHM suppresses caspase-1 activation and limits cytokine maturation [92,93]. This co-ordinated regulation links innates immune receptor signalling with inflammasome activity, providing a unified mechanism for controlling inflammatory amplification. In addition, most evidence for NLRP3 modulation derives from preclinical systems, and the relevance of these findings to human inflammatory diseases remains to be established.

The anti-inflammatory actions of DHM are further reinforced through activation of the Nrf2 pathway, which integrates redox regulation with immune modulation. Nrf2 activation enhances the expression of cytoprotective enzymes while concurrently suppressing inflammatory mediators, highlighting the close interplay between oxidative stress control and inflammatory signalling [104], although Nrf2 activation is a central mechanism, its sustained activation may have context-dependent effects, including potential interference with normal immune responses. In the central nervous system, DHM attenuates neuroinflammation by suppressing microglial activation and promoting a shift from a pro-inflammatory to a neuroprotective phenotype [78,79,105]. This phenotypic modulation reduces the release of neurotoxic cytokines and limits inflammatory damage to neuronal tissues. Similar systemic effects are observed in metabolic disease models, where DHM reduces circulating inflammatory mediators such as IL-1β, IL-6, TNF-α, and MCP-1, while enhancing immunoregulatory cytokines like IL-2 [10,106]. In hepatic inflammation, these effects translate into reduced tissue injury and improved inflammatory profiles [101]. Importantly, variability in experimental models, dosing regimens, and disease contexts complicates direct comparison across studies.

Beyond cytokine regulation, DHM modulates immune cell function. It promotes macrophage polarization toward the anti-inflammatory M2 phenotype while suppressing the pro-inflammatory M1 state, thereby facilitating resolution of inflammation and tissue repair [107]. Additionally, DHM inhibits mast cell degranulation, limiting the release of mediators associated with allergic and inflammatory responses [108]. DHM also influences intracellular signalling networks linked to inflammation. Activation of the AMPK/SIRT1 axis contributes to suppression of NF-κB and AP-1 activity, partly through epigenetic regulation of gene expression [97]. In parallel, inhibition of the JAK/STAT pathway further reduces cytokine-driven inflammatory signalling [10]. In viral infection models, DHM modulates pattern recognition receptor pathways, including TLR3 and TLR9, thereby attenuating excessive cytokine release and limiting immunopathology [88,89,91,109].

**Figure 6:**
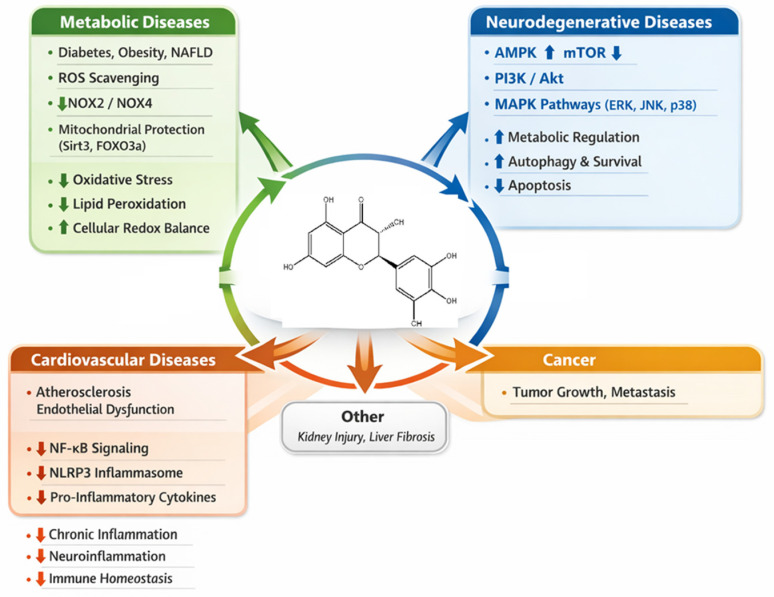
Mechanistic overview of DHM highlighting its integrated regulation of redox homeostasis, kinase-mediated signalling pathways and inflammatory networks.

DHM contributes to the resolution of inflammation associated with metabolic disorders by regulating signalling pathways that link immune responses to metabolic regulation. Activation of the Ca^2+^–CaMKK–AMPK signalling pathway by DHM has been shown to improve insulin sensitivity while simultaneously suppressing inflammation associated with metabolic dysfunction [96]. This dual metabolic and anti-inflammatory action underscores the ability of DHM to address complex disease processes involving both immune dysregulation and metabolic imbalance. These findings demonstrate that DHM exerts multifaceted anti-inflammatory and immunomodulatory effects through coordinated regulation of innate immune receptors, transcription factors, inflammasome activity, and immune cell behaviour. By targeting key signalling pathways such as TLR/NF-κB, NLRP3 inflammasome, Nrf2, AMPK/SIRT1, and JAK/STAT, DHM effectively suppresses excessive inflammatory responses while promoting immune homeostasis. These properties contribute significantly to its therapeutic potential in inflammatory, metabolic, and neurodegenerative diseases as shown in Figure 6.

## 7. Therapeutic Applications of Dihydromyricetin: Integrating Mechanistic Insights for Disease Management

Over the years, DHM has been found to offer therapeutic benefits across several disease categories, making it a compound of immense clinical promise. Its potential therapeutic applications span cardiovascular diseases, metabolic disorders (including diabetes), neurodegenerative diseases, liver diseases, and a variety of inflammation-driven conditions. The therapeutic efficacy of DHM is not merely anecdotal; rather, it is deeply rooted in the molecular and mechanistic actions it exerts within the body, which provide the foundational support for its clinical use [16,110].

### 7.1. Cardiovascular Disease: Preventing Atherosclerosis and Reducing Cardiovascular Risk

Cardiovascular diseases (CVD) remain the leading cause of death globally, driven by the complex interplay of genetic, environmental, and lifestyle factors. Among the most critical contributors to CVD is atherosclerosis, a disease characterized by lipid accumulation and the formation of plaques in the arterial walls. The key risk factors such as high cholesterol, hypertension, and inflammation lead to endothelial dysfunction and the subsequent progression of cardiovascular pathology [111]. This is where DHM shows promise.

Through its potent antioxidant properties, DHM helps to reduce the oxidative stress that is central to atherosclerosis. The flavonoid’s ability to scavenge reactive oxygen species (ROS) reduces oxidative damage in endothelial cells, a key player in the development of cardiovascular disease. Studies have demonstrated that DHM can inhibit the formation of foam cells—a critical step in plaque build-up by improving cholesterol efflux from macrophages and enhancing lipid metabolism. These effects are largely driven by DHM’s modulation of key pathways such as the PPARα and LXRa receptors, which are involved in regulating lipid transport and inflammation in the vascular system [15,38,112].

Furthermore, DHM’s ability to reduce inflammatory cytokines, such as IL-6 and TNF-α, and its inhibition of the NF-κB signalling pathway further solidify its role as a protective agent in CVD. The ability of DHM to modulate these pathways not only prevents plaque formation but also stabilizes existing plaques, reducing the risk of rupture and subsequent cardiovascular events. Thus, by targeting both oxidative stress and inflammation, DHM offers a comprehensive strategy for managing and preventing the progression of atherosclerosis and other cardiovascular disorders [46,113,114].

### 7.2. Metabolic Diseases and Diabetes: Restoring Metabolic Balance

Diabetes mellitus, particularly type 2 diabetes (T2DM), represents a global health crisis, with the World Health Organization projecting a significant increase in prevalence in the coming decades. The disease is characterized by insulin resistance, poor glucose metabolism, and often, dyslipidaemia. These metabolic disturbances, combined with oxidative stress and chronic inflammation, are major contributors to the development of complications such as diabetic nephropathy, retinopathy, and cardiovascular disease [115,116].

DHM offers a multi-pronged approach to combat the metabolic dysfunctions seen in diabetes. Through its modulation of the AMP-activated protein kinase (AMPK) pathway, DHM activates key metabolic processes involved in glucose uptake and lipid oxidation [23]. By activating AMPK, DHM enhances insulin sensitivity and improves glucose metabolism in peripheral tissues, thus aiding in the management of hyperglycaemia and insulin resistance. Moreover, DHM’s ability to regulate the PI3K/Akt pathway further supports its role in enhancing insulin signalling, which is pivotal in diabetes management [117,118,119].

In addition to its effects on glucose metabolism, DHM has been shown to improve lipid profiles by reducing triglyceride and cholesterol levels. This is particularly important for patients with diabetes, who often suffer from dyslipidaemia and have an increased risk of cardiovascular events. DHM’s ability to reduce inflammation and oxidative stress, both of which contribute to the pathogenesis of diabetes and its complications, further enhances its potential as a therapeutic agent for diabetes management.

### 7.3. Neurodegenerative Diseases: Protecting the Brain from Oxidative Damage

Neurodegenerative diseases, including Alzheimer’s disease (AD), Parkinson’s disease, and amyotrophic lateral sclerosis (ALS), are characterized by the progressive degeneration of neurons, often driven by oxidative stress, inflammation, and mitochondrial dysfunction. As the aging population grows, these conditions are becoming more prevalent, leading to a significant burden on healthcare systems worldwide [97,120].

DHM’s neuroprotective effects make it a promising candidate for the management of neurodegenerative diseases. By scavenging free radicals and reducing oxidative stress, DHM helps protect neurons from the damage associated with ROS. Furthermore, DHM’s ability to modulate neuroinflammatory pathways, particularly through its inhibition of NF-κB and NLRP-3 inflammasome activation, offers a means to address the chronic inflammation that exacerbates neurodegeneration [121,122].

Research also suggests that DHM plays a role in improving mitochondrial function, a critical aspect of neuronal health. In neurodegenerative diseases, mitochondrial dysfunction is often observed, contributing to cellular energy deficits and apoptosis. DHM’s ability to regulate mitochondrial dynamics, promote cellular survival, and enhance autophagy further supports its therapeutic application in conditions like AD and Parkinson’s disease. As such, DHM’s antioxidant and anti-inflammatory effects, combined with its potential to restore mitochondrial function, position it as a promising candidate for the treatment of neurodegenerative disorders [123,124,125].

### 7.4. Liver Diseases: Protecting Against Oxidative Damage and Inflammation

Liver diseases, such as non-alcoholic fatty liver disease (NAFLD), alcoholic liver disease (ALD), and cirrhosis, are becoming increasingly common, often exacerbated by metabolic dysfunction, inflammation, and oxidative stress. The liver plays a crucial role in detoxification, lipid metabolism, and immune regulation, and its dysfunction leads to a cascade of health complications [39,126].

DHM’s hepatoprotective effects are primarily driven by its ability to modulate oxidative stress and inflammation within the liver. By reducing ROS levels and improving the function of antioxidant enzymes, DHM helps protect hepatocytes from oxidative damage. Furthermore, DHM’s ability to inhibit the NF-κB pathway and reduce the expression of pro-inflammatory cytokines, such as TNF-α, IL-6, and IL-1β, underscores its anti-inflammatory properties, which are critical in mitigating liver injury and fibrosis [14,127].

In addition to its antioxidant and anti-inflammatory effects, DHM has been shown to improve lipid metabolism in the liver, reducing triglyceride accumulation and inhibiting the formation of hepatic fibrosis. These effects are important for managing liver conditions such as NAFLD, where lipid accumulation and inflammation play central roles in disease progression. Through these mechanisms, DHM holds promise as a therapeutic agent for liver diseases, offering both protective and therapeutic effects [14,128].

### 7.5. Inflammatory Conditions: Modulating Immune Responses

Chronic inflammation is a key driver of many diseases, including autoimmune disorders, arthritis, asthma, and even cancer. DHM’s ability to modulate the immune response and reduce inflammation makes it a valuable therapeutic agent for managing these conditions. By targeting the NF-κB signalling pathway, DHM helps regulate the expression of cytokines and immune mediators involved in inflammation, effectively reducing the immune system’s overactive response [46,129,130].

In autoimmune diseases such as rheumatoid arthritis and inflammatory bowel disease, where the immune system mistakenly targets the body’s own tissues, DHM’s ability to suppress immune cell activation and cytokine production provides a mechanism for alleviating disease symptoms. Furthermore, DHM’s effects on the NLRP-3 inflammasome, a key player in inflammation, provide additional therapeutic potential for managing conditions driven by chronic immune activation [131,132].

Through its diverse biological activities, DHM offers a broad therapeutic spectrum, making it a versatile agent for the management of various chronic diseases. Its anti-inflammatory, antioxidant, and metabolic-regulating effects, driven by the modulation of key molecular pathways, make DHM a promising candidate for addressing the complex pathophysiology of diseases like cardiovascular disorders, diabetes, neurodegenerative conditions, liver diseases, and inflammation-driven conditions. As research continues to uncover the full range of DHM’s mechanistic actions, it is likely that DHM will play an increasingly important role in the clinical management of these diseases, offering new hope for patients suffering from these challenging conditions. Despite broad therapeutic claims, most evidence remains preclinical, with limited standardization in dosing, formulation, and experimental design. This heterogeneity complicates cross-study comparisons and highlights the need for well-controlled clinical investigations. As research continues to uncover the full range of DHM’s mechanistic actions, it is likely that DHM will play an increasingly important role in the clinical management of these diseases, offering new hope for patients suffering from these challenging conditions.

## 8. Pharmacokinetics, Bioavailability, and Metabolic Fate

Despite its promising pharmacological profile, the clinical translation of dihydromyricetin DHM is significantly constrained by unfavourable pharmacokinetic properties, particularly poor bioavailability, limited solubility, and chemical instability. These limitations are largely attributed to its polyhydroxylated flavanonol structure, which, while essential for biological activity, also predisposes the molecule to rapid degradation and extensive metabolism.

DHM contains multiple hydroxyl functional groups that confer strong antioxidant capacity but simultaneously reduce its chemical stability. The compound remains relatively stable under acidic conditions, particularly within a pH range of 1.0–5.0, corresponding to the gastric environment [50]. However, its stability decreases under neutral to slightly alkaline conditions, such as those encountered in the intestine, where partial degradation occurs [133]. These transformations include oxidation, hydrolysis, and structural rearrangements, contributing to the formation of multiple metabolites and reduced availability of the parent compound. Solubility is another critical factor influencing DHM pharmacokinetics. The compound exhibits poor aqueous solubility at room temperature (approximately 0.2–0.32 mg/mL at 25 °C), although solubility improves significantly in hot water and organic solvents such as ethanol [52]. This limited solubility restricts its dissolution in gastrointestinal fluids, thereby impairing absorption following oral administration. Experimental studies using intestinal epithelial models have further demonstrated that DHM permeability is relatively low, although absorption can be modestly enhanced under slightly acidic conditions [33].

Pharmacokinetic studies indicate that DHM is rapidly distributed following oral administration but exhibits limited systemic exposure. Reported oral bioavailability is low, typically below 10%, with values around 4% in some models [134]. After administration, peak plasma concentrations are reached within a relatively short time frame, followed by a rapid decline, reflecting efficient elimination [53]. The elimination half-life generally ranges from several hours, and the compound is largely cleared from the system within approximately 12 h, suggesting minimal risk of long-term accumulation [135]. Metabolism of DHM is complex and involves both host enzymatic systems and gut microbiota. Phase II metabolic processes, particularly glucuronidation and sulphation, contribute to the formation of conjugated metabolites detected in urine [17]. In contrast, intestinal microflora play a significant role in transforming DHM through reduction and dihydroxylation reactions, generating metabolites primarily detected in faecal samples [33]. Notably, a portion of the administered compound is excreted unchanged, indicating incomplete absorption. Overall, multiple metabolites have been identified across biological matrices, reflecting extensive biotransformation.

The gut microbiota has emerged as an important determinant of DHM pharmacokinetics, influencing both its metabolic conversion and bioavailability. Microbial enzymes facilitate structural modifications that may alter biological activity and systemic exposure. This interaction between DHM and intestinal microbiota highlights the need to consider host–microbe interactions when evaluating its pharmacological potential. An important pharmacokinetic feature of DHM is its ability to cross the blood–brain barrier, which supports its observed neuroprotective effects [97]. However, the extent of brain penetration remains limited by its low systemic availability, further emphasizing the need for improved delivery strategies. Toxicological evaluations indicate that DHM possesses a favourable safety profile. Acute and subchronic studies have demonstrated low toxicity, with high tolerated doses and no significant adverse effects observed across a wide dosage range [16]. Long-term feeding studies using DHM-rich plant extracts have similarly shown good safety and even potential immunomodulatory benefits, supporting its suitability for therapeutic development [136].

These pharmacokinetic limitations underscore a major translational bottleneck, as improved bioactivity observed in vitro does not necessarily translate to effective systemic exposure in vivo. To overcome the inherent pharmacokinetic limitations of DHM, various formulation and delivery strategies have been explored. Approaches aimed at enhancing water solubility include nanoparticle-based systems, cyclodextrin inclusion complexes, and co-crystallization techniques [137,138,139]. Alternatively, strategies to increase lipophilicity, such as phospholipid complex formation and chemical modification (e.g., acylation), have been investigated to improve membrane permeability and absorption [140]. Advanced delivery systems, including PEGylated liposomes, chitosan-based nanoparticles, and injectable hydrogel platforms, have shown promise in improving stability, controlled release, and tissue targeting [52,141,142]. While DHM exhibits significant therapeutic potential, its pharmacokinetic limitations; particularly poor solubility, instability, and low bioavailability remain major challenges. Addressing these issues through rational formulation design, structural optimization, and targeted delivery systems will be essential for translating its pre-clinical efficacy into clinical applications.

## 9. Advanced Delivery Systems: Overcoming Limitations

DHM a polyhydroxylated flavanonol, has attracted considerable interest as a multifunctional therapeutic agent due to its ability to regulate redox homeostasis and kinase-mediated signalling pathways [143]. These properties underpin its broad pharmacological effects, including antioxidant, anti-inflammatory, metabolic, and neuroprotective activities. However, despite this promising bioactivity, the clinical translation of DHM remains limited by unfavourable physicochemical and pharmaco-kinetic properties [144].

DHM exhibits poor aqueous solubility, low membrane permeability, and chemical instability. These features contribute to rapid metabolic clearance and extensive first-pass metabolism [145]. As a result, oral administration leads to very low systemic bioavailability, often below 5%, which significantly limits its therapeutic efficacy in vivo [146].

To overcome these challenges, a wide range of advanced delivery strategies has been explored. These include nanoparticle-based systems, liposomal formulations, polymeric micelles, hydrogels, and prodrug approaches [52]. Collectively, these technologies aim to improve solubility, enhance stability, and enable controlled or targeted drug release. More recently, ligand-functionalized and stimuli-responsive systems have been developed to achieve site-specific delivery, thereby increasing therapeutic efficacy while minimizing off-target effects. These advances are critical for translating DHM from preclinical research into clinical application [147].

### 9.1. Nanoparticle-Based Delivery Systems

Nanoparticle-based formulations represent one of the most effective strategies for improving DHM pharmacokinetics. Polymeric nanoparticles, solid lipid nanoparticles (SLNs), and nanostructured lipid carriers (NLCs) have been widely investigated for their ability to enhance solubility, stability, and delivery efficiency [52,148].

**Figure 7:**
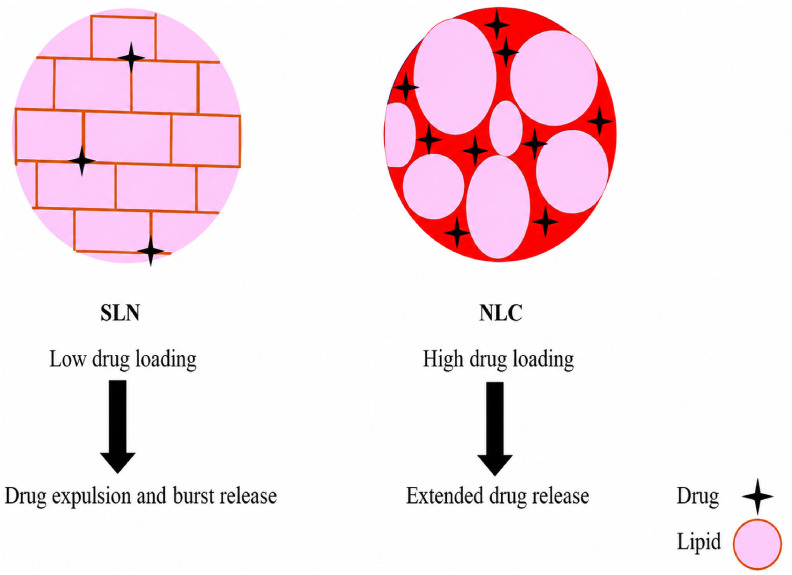
Schematic view of the solid lipid nanoparticle (SLN) and nanostructured lipid carriers (NLCs) showing the drug location within the lipid matrix. With permission from Wolters Kluwer [148].

Polymeric nanoparticles, particularly those based on poly(lactic-co-glycolic acid) (PLGA), offer excellent biocompatibility and controlled drug release [149]. These systems can be surface-modified with targeting ligands or polymers, enabling improved cellular uptake and tissue specificity as illustrated in Figure 7. This is especially relevant for directing DHM to organs such as the liver or brain, where its biological activity is most pronounced [150].

Lipid-based nanoparticles provide complementary advantages. SLNs protect DHM from gastrointestinal degradation and improve oral absorption [151]. NLCs, which incorporate both solid and liquid lipids, offer higher drug loading capacity and improved release profiles. These systems are particularly effective for solubilizing poorly water-soluble compounds, resulting in enhanced systemic exposure [152,153].

Formulation parameters such as particle size, surfactant concentration, and processing conditions play a critical role in determining nanoparticle performance. Particle sizes below 200 nm are generally optimal, as they promote efficient cellular uptake and prolonged circulation [154,155,156]. Notably, DHM-loaded chitosan nanoparticles have demonstrated improved bioavailability and enhanced activation of antioxidant pathways, highlighting both pharmacokinetic and pharmacodynamic benefits [157].

This underscores the dual benefit of encapsulation, improving pharmacokinetic performance while simultaneously augmenting pharmacodynamic efficacy. Nanoparticle-based delivery systems represent a transformative approach to DHM therapeutics, bridging the gap between its potent bioactivity and clinical applicability [158]. By enhancing solubility, protecting against premature degradation, and enabling targeted release, these platforms significantly expand the translational potential of DHM in managing oxidative stress-driven and kinase-mediated pathologies [159].

### 9.2. Liposomal Formulations and Liposome-Composite Hydrogels

Liposomes are spherical vesicles composed of one or more phospholipid bilayers and represent a well-established drug delivery platform. Their amphiphilic structure allows simultaneous encapsulation of hydrophilic and lipophilic compounds, while their surface can be modified to optimize pharmacokinetics and biodistribution. For DHM, liposomes improve solubility, enhance cellular uptake, and protect against oxidative degradation.

However, conventional liposomes face limitations, including rapid clearance, in-stability in biological fluids, and susceptibility to uptake by the mononuclear phagocyte system (MPS) [107]. To address these challenges, several modifications have been developed. PEGylation, for example, introduces a hydrophilic “stealth” layer that reduces immune recognition and prolongs circulation time. This modification significantly improves DHM bioavailability and systemic exposure [160].

In addition, ligand functionalization enables active targeting. Liposomes conjugated with folic acid, peptides, or antibodies can selectively accumulate in tumour or inflamed tissues. This enhances site-specific delivery and reduces off-target effects [161]. A more recent advancement involves liposome–hydrogel composite systems. In these formulations, liposomes are embedded within biocompatible hydrogels such as gelatin, chitosan, or methacrylated hyaluronic acid. These systems combine the encapsulation efficiency of liposomes with the sustained release properties of hydrogels. As a result, they improve stability, protect DHM from degradation, and enable controlled drug release [162,163] as presented in Table 2.

### 9.3. Cyclodextrin Inclusion Complexes and Cocrystals

Cyclic oligosaccharides characterized by their hydrophobic internal cavities and hydrophilic outer surfaces, have proven to be highly effective in enhancing the solubility and stability of poorly water-soluble compounds such as DHM [168]. Cyclodex-trins (CDs) are cyclic oligosaccharides with hydrophobic cavities that can encapsulate poorly soluble compounds. Inclusion complex formation significantly enhances DHM solubility and stability by shielding it from degradation. β-cyclodextrin and hydroxy-propyl-β-cyclodextrin have been shown to increase DHM solubility by more than 50-fold [169].

Advanced CD-based systems, including nanofibers and nanosponges, further improve delivery performance. Nanofibers offer rapid dissolution due to their high surface area, while nanosponges provide controlled release through porous polymeric networks [170,171]. These systems are particularly advantageous for oral delivery [52].

In parallel, cocrystal engineering has emerged as a complementary strategy to overcome the limitations of DHM’s crystalline structure. DHM possesses an extensive hydrogen bonding network that contributes to its poor solubility and slow dissolution [172]. By co-crystallizing DHM with pharmaceutically acceptable conformers such as caffeine, urea, triethanolamine (TEA), or calcium salts this rigid hydrogen bonding lattice is disrupted, resulting in improved physicochemical properties [173]. Notably, DHM-TEA and DHM-Ca cocrystals exhibit 4–6-fold higher solubility compared to native DHM, alongside significantly enhanced in vivo absorption [174].

These improvements translate into superior pharmacodynamic activity, including more pronounced antioxidant and anti-inflammatory effects. The dual strategies of cyclodextrin complexation and cocrystal engineering thus represent powerful tools for modulating the physicochemical and pharmacokinetic profile of DHM. Cyclodextrins provide versatile, biocompatible carriers that enhance solubility and stability, while cocrystals offer a rational design approach to fundamentally alter DHM’s crystalline architecture [175]. Together, these innovations expand the translational potential of DHM, enabling its progression from preclinical promise to clinically viable formulations.

### 9.4. Prodrug and Chemical/Enzymatic Modification Strategies

Chemical modification of DHM provides an effective approach to overcoming its pharmacokinetic limitations. Enzymatic glucosylation significantly improves water solubility, with some derivatives showing up to 600-fold increases compared to the parent compound. This modification enhances oral absorption and systemic exposure [140].

Acylation with fatty acids increases lipophilicity and membrane permeability. Such derivatives may facilitate blood–brain barrier penetration, which is critical for neuroprotective applications [176]. These modifications are typically achieved using regioselective enzymatic processes, ensuring precise structural control.

This modification is particularly relevant for DHM’s neuroprotective applications, where efficient CNS delivery is critical. [140] demonstrated that acylated DHM derivatives retained antioxidant activity while exhibiting improved permeability and stability, highlighting their promise for neurological indications. These modifications are typically achieved through regioselective biocatalysis, employing glycosyltransferases or lipases to ensure precise substitution patterns [140]. The resulting derivatives display distinct solubility, permeability, and antioxidant pro-files, enabling application-specific optimization. For instance, glucosylated DHM analogues are particularly suited for oral nutraceutical formulations, while acylated derivatives may be better positioned for CNS-targeted therapies [177].

Prodrug strategies further extend this paradigm by temporarily masking DHM’s reactive hydroxyl groups with metabolically labile moieties. Upon enzymatic cleavage in vivo, the parent DHM is released in a controlled manner, thereby improving pharmacokinetics and reducing premature metabolism. Such prodrug approaches have been proposed to balance DHM’s dual requirements of systemic stability and site-specific activation [178,179].

### 9.5. Microemulsions, Self-Emulsifying Drug Delivery Systems (SEDDS), and Nanoemulsions

Emulsion-based delivery systems have emerged as versatile and clinically translatable platforms for enhancing the pharmacokinetic performance of DHM. By lever-aging the physicochemical principles of oil–water dispersion, these systems generate nanostructured carriers that significantly improve solubility, dissolution, and absorption, thereby addressing DHM’s intrinsic limitations of poor aqueous solubility and low oral bioavailability [180].

Microemulsions are thermodynamically stable, isotropic mixtures of oils, surfactants, and co-surfactants that spontaneously form fine oil-in-water dispersions upon contact with gastrointestinal fluids [181]. The resulting nanodroplets, typically <100 nm in diameter, provide a large interfacial area for drug absorption, thereby accelerating dissolution kinetics and enhancing intestinal uptake [182]. In animal models, DHM-loaded microemulsions have demonstrated 4–5-fold increases in oral bioavailability, highlighting their potential to bridge the gap between preclinical efficacy and clinical translation. Self-Emulsifying Drug Delivery Systems (SEDDS) extend this concept by employing pre-formulated isotropic mixtures that undergo rapid emulsification in the gastrointestinal tract without external energy input [183,184].

Upon exposure to digestive fluids, SEDDS formulations generate uniform nanodroplets that not only enhance solubilization but also protect DHM against enzymatic and acidic degradation, thereby improving systemic exposure. Zhang et al., (2022) reported that DHM-loaded SEDDS achieved markedly higher plasma concentrations compared to conventional suspensions, underscoring their promise for oral nutraceutical and pharmaceutical applications [52].

Nanoemulsions provide additional stability and can be tailored for controlled release. These systems improve gastrointestinal absorption and prolong circulation time, contributing to more consistent pharmacokinetic profiles [185,186]. Encapsulation within gelatin or polymeric capsules provides additional protection against gastric degradation, ensuring that DHM reaches systemic circulation in its active form [187].

### 9.6. Hydrogels and Implantable Systems

Hydrogels, three-dimensional networks of cross-linked hydrophilic polymers, have emerged as highly versatile platforms for drug delivery owing to their biocompatibility, tuneable mechanical properties, and capacity for local, sustained, or stimuli-responsive release. Their ability to retain large amounts of water while maintaining structural integrity makes them particularly suitable for encapsulating hydrophilic compounds such as DHM, thereby addressing its poor solubility and rapid clearance [52].

Recent advances have focused on engineering smart hydrogels that respond to physiological cues, enabling controlled and site-specific DHM release. Thermoresponsive hydrogels, exploit temperature-sensitive polymers (e.g., poly(N-isopropylacrylamide)) to release DHM in response to local thermal changes, useful in inflamed or tumour tissues where temperature is elevated [188]. pH-sensitive hydrogels are designed to swell or degrade under acidic conditions, aiding DHM re-lease in the gastrointestinal tract or tumour microenvironments [189].

Redox-responsive hydrogels, incorporate disulphide linkages that cleave under oxidative stress, enabling DHM release precisely where ROS levels are pathologically elevated. Multi-responsive hydrogels, combine two or more triggers (e.g., pH + redox) to achieve highly selective release profiles, maximizing therapeutic efficacy while minimizing systemic exposure [190].

### 9.7. Protein-Based and Pickering Emulsion Systems

Protein–polyphenol complexes have emerged as promising food-grade delivery vehicles for DHM, particularly within the nutraceutical and functional food sectors. Proteins such as soy protein isolate (SPI), whey protein, and casein can interact with DHM through hydrogen bonding, hydrophobic interactions, and electrostatic forces, forming stable amorphous matrices that enhance solubility, dispersion, and bioaccessibility [191,192].

**Figure 8:**
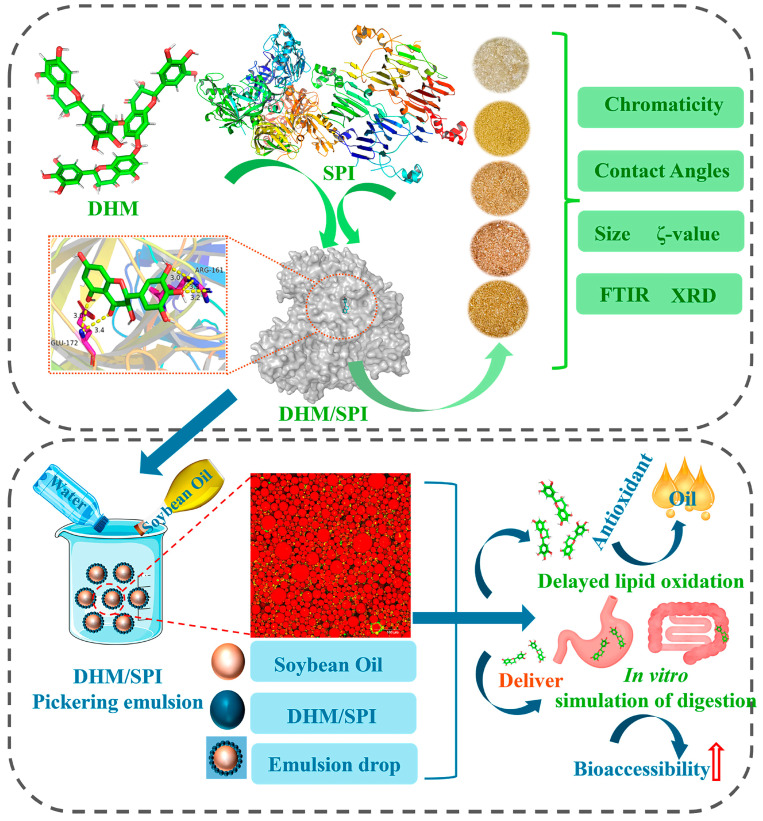
Formation and Functional Application of DHM/SPI-Based Pickering Emulsions [193].

Another interesting process is the pickering emulsions, which is stabilized by solid particles rather than conventional surfactants, offer additional advantages. DHM/SPI Pickering emulsions form elastic, gel-like network structures that encapsulate DHM, protecting it from degradation during gastrointestinal transit [193], illustrated graphically in Figure 8. In vitro digestion models have demonstrated that such systems achieve bioaccessibility levels exceeding 33%, a significant improvement compared to unformulated DHM. These results highlight the potential of protein-based emulsions to serve as safe, scalable, and consumer-friendly delivery platforms, aligning with the growing demand for functional foods enriched with bioactive flavonoids [194].

### 9.8. Targeted and Stimuli-Responsive Delivery Strategies

The therapeutic versatility of DHM can be further enhanced through advanced targeting and stimuli-responsive delivery systems. These are achieved through processes such as Ligand-mediated targeting, where nanoparticles and liposomes functionalized with ligands such as folic acid, RGD peptides, aptamers, or monoclonal antibodies enable selective accumulation in tumours or inflamed tissues, exploiting receptor-mediated uptake pathways [195].

Another is the stimuli-responsive systems, Smart nanocarriers release DHM in response to specific environmental triggers, including pH gradients (tumour microenvironment), redox potential (oxidative stress), enzymatic activity (protease-rich tissues), or external stimuli (light, ultrasound). Also, precision medicine potential, strategies allow DHM to act in a spatiotemporally controlled manner, minimizing off-target effects and maximizing therapeutic efficacy. Such approaches are particularly relevant in oncology and chronic inflammatory diseases, where targeted delivery is essential for clinical success [196].

### 9.9. Blood–Brain Barrier (BBB) Delivery Strategies

For DHM’s neuroprotective applications, effective delivery across the blood–brain barrier (BBB) remains a critical challenge. Several innovative strategies have been explored such as Surface modification of nanocarriers, which deals with decorating nanoparticles or liposomes with transferrin, mannose, or cell-penetrating peptides facilitating receptor-mediated transcytosis across the BBB [197].

Exosome-based delivery, harnessing naturally occurring extracellular vesicles, provides biocompatible carriers with intrinsic BBB-crossing capabilities, reducing immunogenicity and enhancing CNS uptake [198]. Intranasal administration, utilizes direct nose-to-brain delivery bypasses systemic circulation and first-pass metabolism, offering rapid CNS uptake and improved therapeutic efficacy. Preclinical studies have demonstrated that these approaches significantly enhance brain accumulation of DHM, improving outcomes in models of Alzheimer’s disease, Parkinson’s disease, and ischemic injury. These findings underscore the potential of BBB-targeted strategies to unlock DHM’s full neuroprotective promise [199].

While these advanced delivery strategies show clear promise, most remain at the proof-of-concept stage, with limited in vivo validation and minimal clinical translation to date.

## 10. Safety, Toxicological Profile, and Translational Considerations

### 10.1. Preclinical Safety and Toxicity Data

Acute and chronic toxicity, DHM exhibits very low acute toxicity in animal models. The reported LD_50_ in rats is >10 g/kg, while estimated safe doses in mice and humans are 16 g/kg and 1.6 g/kg, respectively, indicating a wide therapeutic window [200,201]. Oral administration of 150–1500 mg/kg in rats produced no significant changes in body weight, temperature, or organ histology, underscoring its benign safety profile [202]. Cytotoxicity, in vitro studies confirm that DHM is non-cytotoxic to normal human cell lines, including hepatocytes (LO-2), prostate epithelial cells (PrEC), and mammary epithelial cells (MCF-10A). However, carrier-related cytotoxicity must be carefully evaluated in advanced formulations. For instance, polymers such as Eudragit RS100^®^ (Evonik Operations GmbH, Darmstadt, Germany) have been associated with DNA damage unless mitigated by DHM inclusion, highlighting the importance of excipient selection in nanocarrier design [203]. Standard assays have reported no evidence of genotoxicity or carcinogenicity. Nevertheless, long-term studies remain warranted to confirm these findings, particularly for chronic nutraceutical or pharmaceutical use [204]. DHM and its delivery systems (liposomes, hydrogels, cyclodextrins) are generally non-immunogenic and well tolerated. However, the immunogenicity of novel nanocarriers or targeting ligands should be assessed on a case-by-case basis to ensure translational safety [205].

### 10.2. Clinical Data and Human Trials

Human data remain limited but promising. A double-blind, randomized, placebo-controlled trial in 60 patients with non-alcoholic fatty liver disease (NAFLD) demonstrated significant improvements in liver enzymes, glucose and lipid metabolism, and inflammatory markers with DHM supplementation (2 × 150 mg capsules, twice daily for three months). Another trial in 80 patients with type 2 diabetes mellitus (T2DM) reported improved glycaemic control and metabolic markers [206]. A first-in-human Phase I, dose-escalation study (NCT05623501) is underway to assess the safety, pharmacokinetics, and maximum tolerated dose of DHM in healthy volunteers, with doses ranging from 300 to 900 mg (with or without L-lysine) administered as oral solutions. Preliminary results indicate good tolerability and no serious adverse events [206]. DHM is currently marketed as a dietary supplement in several countries and is recognized as a “new resource food” by the Chinese Ministry of Health. However, it is not yet approved as a pharmaceutical drug, and regulatory pathways for novel formulations (e.g., nanocarriers, prodrugs) require comprehensive safety and efficacy data.

### 10.3. Translational and Manufacturing Considerations

Many advanced delivery systems (nanoparticles, liposome-hydrogel microspheres, cyclodextrin complexes) involve complex manufacturing processes, expensive excipients, and challenges in scalability and reproducibility. Simplification of protocols, use of GRAS (generally recognized as safe) materials, and robust quality control are essential for clinical translation. DHM is chemically unstable under neutral/alkaline pH, elevated temperatures, and in the presence of metal ions or light. Formulations should maintain an acidic environment, minimize exposure to oxidants, and incorporate stabilizers (e.g., ascorbic acid, chelating agents) to preserve potency during storage and administration. Accurate quantification of DHM in formulations and biological matrices is critical for pharmacokinetic and quality control studies. Validated methods include HPLC-DAD, LC-MS/MS, and UV-Vis spectrophotometry, each offering specific advantages for sensitivity, selectivity, and throughput. Standardization of DHM content and purity is necessary to ensure batch-to-batch consistency. Transitioning DHM from preclinical promise to clinical application requires adherence to Good Manufacturing Practice (GMP), comprehensive toxicological evaluation, and compliance with regulatory guidelines for novel excipients and delivery systems. Ethical considerations include informed consent, risk–benefit assessment, and post-marketing surveillance for adverse events.

## 11. Current Limitations and Future Perspectives

### 11.1. Current Limitations

Despite significant advances in the understanding and formulation of DHM, several critical limitations persist that hinder its clinical translation. DHM preparations often suffer from batch-to-batch variability in purity, content, and bioactivity, particularly in dietary supplements and herbal extracts, compounded by the presence of multiple isomers (due to two chiral centres) and degradation products that complicate standardization. Advanced delivery systems, while effective in preclinical models, face formulation and manufacturing barriers, including scalability, reproducibility, regulatory approval, and reliance on expensive or potentially toxic excipients. Bioavailability and stability remain major challenges, as maintaining supersaturation and preventing precipitation or degradation in vivo is difficult, with cocrystals and inclusion complexes prone to dissociation under physiological conditions. Furthermore, the structure–activity relationships (SAR) of DHM and its derivatives are incompletely characterized, limiting rational drug design and optimization of kinase-targeted therapies. Finally, DHM’s multi-target nature introduces mechanistic complexity, necessitating systems-level approaches to fully understand its holistic effects and potential synergistic interactions with other therapeutics.

### 11.2. Future Perspectives

To overcome these limitations, future research should prioritize the development and validation of robust analytical methods (HPLC, LC-MS/MS, NMR) for quality control, alongside the isolation and characterization of individual DHM isomers to assess their distinct pharmacological profiles and enable monoisomeric drug preparations. Formulation science must advance toward cost-effective, scalable, and environmentally friendly strategies, leveraging GRAS materials, green chemistry approaches (e.g., electrospinning for nanofibers), and modular design principles to streamline manufacturing and facilitate regulatory approval. Delivery systems should be optimized to sustain supersaturation, enhance permeability, and protect DHM from degradation, incorporating novel excipients, stabilizers (ascorbic acid, chelators), and encapsulation techniques to improve pharmacokinetics. Systematic SAR studies, high-throughput screening, and rational design of DHM analogues guided by molecular docking will enable the identification of derivatives with optimized kinase inhibition, redox modulation, and pharmacokinetic properties. A critical limitation across the DHM literature is the predominance of in vitro and small-animal studies, often employing supraphysiological concentrations. Furthermore, variability in experimental design, disease models, and outcome measures reduces reproducibility and comparability. Future research should prioritize standardized protocols, pharmacokinetic–pharmacodynamic integration, and well-designed clinical studies to validate the therapeutic potential of DHM. Finally, the integration of systems biology and network pharmacology approaches combining omics data, computational modelling, and experimental validation will illuminate DHM’s multi-target interactions, inform combination therapy strategies, and support precision medicine applications.

## 12. Conclusions

Dihydromyricetin (DHM) stands at the forefront of natural product-based therapeutics, distinguished by its unique capacity to modulate redox homeostasis and kinase-driven signalling pathways implicated in diverse disease processes. Its antioxidant, anti-inflammatory, neuroprotective, metabolic, and anticancer effects are mediated through the orchestration of key molecular networks, including Nrf2/HO-1, NF-κB, MAPK, PI3K/Akt, AMPK/mTOR, and SIRT1 axes. These pleiotropic actions position DHM as a prototypical multi-target agent capable of addressing the complex pathogenesis of chronic diseases. However, the clinical translation of DHM has been historically constrained by poor aqueous solubility, low membrane permeability, chemical instability, and rapid metabolism, culminating in suboptimal bioavailability. The advent of advanced delivery systems—encompassing nanoparticles, liposomes, hydrogels, cyclodextrin inclusion complexes, cocrystals, and prodrug strategies—has revolutionized the pharmacokinetic landscape of DHM, enabling enhanced absorption, stability, and targeted delivery to disease-relevant tissues. These innovations have not only improved therapeutic efficacy but also minimized systemic toxicity and dosing frequency. Preclinical and emerging clinical data affirm the safety and tolerability of DHM, with a wide therapeutic window and minimal adverse effects. Nevertheless, challenges remain in the standardization of formulations, elucidation of precise mechanisms of action, and validation of efficacy in large-scale human trials. The potential for drug–drug interactions, particularly via CYP450 and OATP pathways, warrants careful consideration in polypharmacy contexts. Looking forward, the integration of systems biology, omics technologies, and network pharmacology will be instrumental in unravelling the complex interactome of DHM and guiding rational drug design. Structure–activity relationship studies and kinase-targeted modifications offer avenues for the development of next-generation DHM derivatives with tailored pharmacological profiles. The convergence of multidisciplinary research efforts, coupled with advances in analytical and manufacturing technologies, is poised to accelerate the clinical translation of DHM-based therapeutics.

## Figures and Tables

**Table 1:** Molecular Targets and Mechanisms of Dihydromyricetin.

Disease/Model	Target Pathway	Key Proteins	Biological Outcome	Experimental Model	Refs.
Cardiovascular (DOX-induced cardiotoxicity)	AMPK/mTOR	AMPK ↑, mTOR ↓	Reduced apoptosis and oxidative stress; enhanced autophagy	H9c2 cardiomyoblasts (rat, in vitro); C57BL/6 mice treated with doxorubicin (in vivo)	[70]
Atherosclerosis/Endothelial dysfunction	PI3K/Akt/Nrf2	Akt ↑, Nrf2 ↑, HO-1 ↑, eNOS ↑	Reduced ROS, improved endothelial function, anti-apoptosis	HUVECs (human umbilical vein endothelial cells, in vitro), ApoE^−^/^−^ mice (in vivo)	[71,72,73]
Oxidative stress	Nrf2/ARE	Nrf2 ↑, Keap1 ↓, HO-1 ↑, SOD ↑	Enhanced antioxidant defense, reduced lipid peroxidation	HepG2, H9c2 cells (in vitro); C57BL/6 mice (in vivo)	[62,68]
Neurodegeneration (AD model)	AMPK/SIRT1	AMPK ↑, SIRT1 ↑, Bax ↓, Bcl-2 ↓	Reduced neuronal apoptosis, improved cognition	Aβ-induced mice (in vivo)	[74,75]
Neurodegeneration (PD model)	AMPK/ULK1	AMPK ↑, ULK1 ↑	Enhanced autophagy, reduced α-synuclein aggregation	MPTP-induced C57BL/6 mice (in vivo)	[76]
Neuroinflammation	NF-κB/NLRP3	NF-κB p65 ↓, NLRP3 ↓, caspase-1 ↓, IL-1β ↓	Suppressed microglial activation and inflammasome activity	LPS-induced mice (in vivo)	[77,78,79]
Metabolic disorders (insulin resistance)	AMPK/GLUT4	AMPK ↑, GLUT4 ↑	Increased glucose uptake, improved insulin sensitivity	3T3-L1 adipocytes (in vitro); high-fat diet-induced mice (in vivo)	[80,81]
Diabetes/glucose metabolism	PI3K/Akt	PI3K ↑, Akt ↑, GSK-3β ↓	Enhanced glucose utilization and glycogen synthesis	STZ-induced rats (in vivo)	[82,83]
Obesity-related inflammation	AMPK/SIRT1/NF-κB	AMPK ↑, SIRT1 ↑, NF-κB ↓	Reduced inflammatory cytokines, improved metabolic balance	High-fat diet-induced C57BL/6 mice (in vivo)	[10]
NAFLD	AMPK/MAPK/NF-κB	AMPK ↑, MAPK ↓, NF-κB ↓	Reduced lipid accumulation and hepatic inflammation	MCD diet-induced mice (in vivo)	[84]
Diabetic nephropathy	PI3K/Akt/mTOR	PI3K ↑, Akt ↑, mTOR ↓, PTEN ↓	Enhanced autophagy, reduced fibrosis	STZ-induced mice (in vivo)	[83]
Liver fibrosis	PI3K/Akt/NF-κB	Akt ↑, NF-κB ↓, TGF-β1 ↓	Reduced inflammation and apoptosis	CCl4-induced mice (in vivo)	[85]
Acute kidney injury	MAPK/NF-κB/Nrf2	p38 ↓, JNK ↓, NF-κB ↓, Nrf2 ↑	Reduced oxidative stress, inflammation, ferroptosis	Cisplatin-induced mice (in vivo)	[86]
Viral infection (ASFV, influenza)	TLR/MAPK/NF-κB	TLR4 ↓, MyD88 ↓, MAPK ↓, NF-κB ↓	Reduced cytokine release, inhibited viral replication	PAMs (ASFV, in vitro); MDCK cells (influenza, in vitro)	[46,87,88,89]
Inflammation (general)	TLR4/NF-κB	TLR4 ↓, MyD88 ↓, IκBα ↑, p65 ↓	Decreased TNF-α, IL-6, IL-1β, COX-2	RAW 264.7 macrophages (in vitro); LPS-induced mice (in vivo)	[71,90,91]
Inflammasome activation	NLRP3 pathway	NLRP3 ↓, caspase-1 ↓, IL-1β ↓, IL-18 ↓	Suppressed inflammasome activation	THP-1 macrophages (in vitro); LPS/ATP-induced mice (in vivo)	[92,93]
Cancer (colon cancer)	AMPK/MAPK (JNK/p38)	AMPK ↑, JNK ↑, p38 ↑, XAF1 ↑	Induced apoptosis via ER stress	HCT116, HT29 cells (in vitro)	[94]
Cancer (osteosarcoma)	TNF-α/p38 MAPK/MMP-2	p38 ↓, MMP-2 ↓	Reduced invasion and metastasis	MG-63, U2OS cells (in vitro)	[95]
Insulin resistance (inflammation-induced)	PLC–CaMKK–AMPK	PLC ↑, CaMKK ↑, AMPK ↑	Improved insulin sensitivity	C2C12 myotubes (in vitro); high-fat diet-induced mice (in vivo)	[96]

**Table 2:** Representative Liposome-Composite Hydrogel Microsphere (LHM) Delivery Systems.

System Name	Liposome Structure	Hydrogel Matrix	Payload	Size (nm/μm)	Functionality	Application	Refs.
ChsMA@Lipo	HSPC, mPEG2000-DSPE, cholesterol	ChsMA, Chs, sodium alginate	Liquiritin	122/220	Controlled-release, targeting	Osteoarthritis	[162]
MELs	PC, cholesterol, DPPC	Poly(L-lysine), alginate	HBsAg	50–800/400	Controlled-release, protection	Vaccine delivery	[164]
PPD-Lipo@HMs	HSPC, egg yolk lecithin	Bletilla striata polysaccharide	20(S)-protopanaxadiol	118/332	Microenvironment response	Diabetic wound repair	[165]
GEF-loaded liposome gel beads	S80, DPPC	Sodium alginate	Gefitinib	686–712	Controlled-release, adaptation	IP administration	[162]
GM@PDA@Lipo-Ebselen	Cholesterol, lecithin	Gelatin, methacrylic anhydride, polydopamine	Ebselen	141/96–97	Controlled-release, adhesion	Hearing impairment	[166]
ChSMA-RGD microspheres	HSPC, DOPE, cholesterol, octadecylamine	ChsMA, LAP, EFL	TGF-β1	178/118	Lubrication, protection	Osteoarthritis	[167]
Cur-R-CCMBs	Phospholipids, rhamnolipids	Chitosan, κ-carrageenan	Curcumin	116/—	Controlled-release	Chronic wound infection	[162]
AST NSC/HSA-PEG Liposomes @SA/CMCS Microspheres	Cholesterol, lecithin, NSC, HSA, AST	SA, CMCS	Astaxanthin	83/—	pH responsive, controlled release	Hypercholesterolemia	[58]
E7-Lipo@Alg/Cs	E7-peptide, lecithin, DSPE-PEG2K-NHS	Alginate, chitosan	Fisetin	153/320	Targeted, protection, controlled-release	Osteoporosis	[58]

## Data Availability

The original contributions presented in this study are included in the article. Further inquiries can be directed to the corresponding author.
